# Learning in brain and machine—complexity, Gödel, Aristotle

**DOI:** 10.3389/fnbot.2013.00023

**Published:** 2013-11-27

**Authors:** Leonid Perlovsky

**Affiliations:** Athinoula A. Martinos Center for Biomedical Imaging, Harvard UniversityCharlestown, MA, USA

**Keywords:** learning, complexity, algorithms, cognition, combinations, logic, logical bias

## Combinatorial complexity of learning

Brains learn much better than computers, this has been discussed in a number of reviews on artificial intelligence, pattern recognitions, and neural networks (Perlovsky, 2001, 2006a). But why? Is there a fundamental reason behind computers being slow learners? Often slow learning is discussed in terms of computational complexity (Perlovsky, 1998), which is usually measured by the number of operations. Scientists have thought that faster computers would be able to catch up with the brain. Still, this has not happened despite computers becoming increasingly faster. Reviews (Perlovsky, 2001, 2006a; Perlovsky et al., 2011) have explained why: computational complexity of learning algorithms grows as a combinatorial (exponential) function of the complexity of a problem to be learned. This means that a learning algorithm might look like it's quite capable of learning, and indeed, it learns solutions to simple problems. However, slightly more complex problems require not just slightly more computations, but require significantly more. So much more, in fact that learning problems of average complexity require more learning examples and more computer operations than all of the interactions of all elementary particles in the entire life of the Universe (In this article such complexity is called “practically infinite.”).

The reason for combinatorial complexity can be explained as follows: consider first, an example of a simple problem requiring no combinatorial complexity for learning: recognition of a single isolated object, which always appears exactly the same. Learning consists in storing in memory the object's image. Recognition consists in matching the stored image to a newly presented image: match or no match. The complexity of this algorithm approximately equals the number of pixels in an image. But in a real situation the object is not always exactly same; the algorithm has to account for variations in viewing angles, distance, color, etc. In addition, other objects are present with their variabilities. Combinations of various objects with their variabilities lead to combinatorial complexity. Combinations of all pixels in the field of view should be considered. A human eye senses ~10,000 pixels 10 times a second. Today, sensors measure millions of pixels each second (or more). The number of combinations of these pixels is “practically infinite”; combinations of 100 pixels (a relatively simple problem) are 100^100^; this number is close to all of the interactions of all elementary particles in the entire life of the Universe.

## Gödel theory and combinatorial complexity

Before considering how brains perceive objects, let us consider a parallel to the above complexity problem: the Gödel theory (Gödel, 2001). Following (Penrose, 1994) it can be described as a proof that the collection of all logical statements must include unprovable statements, and therefore, there is no complete logical basis for mathematics. Gödel's theory received critical acclaim upon its publication in 1931; in 2000 the New York Times listed the Gödel theory along with the theory of relativity among the greatest scientific achievements of the 20th century. However, the consequences of this theory outside of mathematical logic and the philosophy of mathematics are limited. With one exception: the Lucas–Penrose argument (Lucas, 1961; Penrose, 1994), which suggests that the mind is not a formal logical system. Nevertheless, for decades artificial intelligence has attempted to develop formal logical models of the mind, and these attempts continue today. Apparently, the consequences of the Gödel theory have not been appreciated. This article discusses the much *wider significance* of the Gödel theory for modeling the mind, as well as for machine learning in general.

In developing his theory Gödel demonstrated that all logical statements are equivalent in some way to all sequences of zeros and ones. It was essential for Gödel to consider infinite sequences of zeros and ones. The entire collection of such sequences is infinite and contains all infinite combinations of zeros and ones. The number of such combinations is a *continuum*, *a “larger” infinity* than the countable infinity of the initial sequences. If we limit the sequences to finite ones, say, to the length *N*, they contain combinations of zeros and ones of length *N*, and their number is 2*^N^*. In particular, if *N* = 300 (not a very large number) the number of sequences is ~100^100^, the “practically infinite” number discussed above. In both cases of finite and infinite sequences the number of combinations turns out to be significantly larger than the original sequence length. If Gödel's arguments are applied to any finite system, such as a computer, or a brain, and only finite combinations are considered, Gödel's proof of the existence of unprovable statements would not stand. A different difficulty would be faced, the practically infinite number of possible statements. No system, the mind or a computer would ever be able to count these statements; no algorithm would be able to execute so many operations. Similar to the Gödelian case, the number of combinations is “much larger” than the initial complexity. The combinatorial complexity of the logical algorithms considered previously is related to Gödel's argument when applied to a finite system.

As discussed above, machine learning and mathematical models of the mind face algorithmic difficulties related to combinatorial complexity. These difficulties are related to the use of logic in algorithms similar to the existence of unprovable statements in the Gödel theory. We face the possibility that the combinatorial complexity encountered since the 1950s is of similar fundamental origin as the Gödel theory, the fundamental limitation of logic.

## How the mind perceives objects

Nevertheless, the mind works, visual systems perceive objects. To understand mathematically how this is possible, mathematicians have to consider the consequences of the Gödel theory and the Lucas–Penrose argument in full honesty. The mind is not a logical system. To make machines capable of learning and to model mathematically the learning abilities of the mind, new types of algorithms are needed that avoid combinatorial complexity. Several mathematical approaches have been proposed to overcome the limitations of logic; however, learning algorithms still have to use logical statements as a part of learning (Perlovsky, 2001; Perlovsky et al., 2011) and combinatorial complexity cannot be avoided. Logic limitations have been overcome in dynamic logic (Perlovsky, 2006b; Perlovsky et al., 2011; Kovalerchuk et al., 2012), which is not a collection of static statements but a process-logic. These processes evolve from vague-fuzzy states to logical states (Perlovsky et al., 2011). Whereas a logical state corresponds to an individual property or object and their combinations have to be considered, a vague state corresponds to a multiplicity of properties and objects, and no combinations have to be considered. Logical states appear only at the end of the dynamic logic processes when a learning problem is solved.

Dynamic logic algorithms model uncertainty by using similarity functions among representations of concepts and incoming data. Often, these similarity functions are modeled functionally similar to probability densities. The dynamic logic idea “from vague-to-crisp” is implemented by initiating probability density functions with large variances. In the iterative dynamic logic processes variances might be reduced to small values, resulting in logic-like very narrow pdfs.

Dynamic logic algorithms have overcome the limitations of logic, have solved previously unsolvable problems, and have not only reached, but exceeded the performance of the human mind (Perlovsky, 2010). Brain imaging experiments have demonstrated that dynamic logic and its vague-to-crisp processes are adequate models for actual brain-mind perception processes (Bar et al., 2006; Perlovsky, 2009). Perception in the brain works fast by matching top-down and bottom-up signals via evolving vague “top” mental representations into logical (or nearly so) representations matching sensory data, while avoiding combinatorial complexity.

## Conscious and unconscious mental operations, logical bias

Brain imaging experiments demonstrating the vague-to-crisp perception in neural mechanisms (Bar et al., 2006) have also demonstrated that vague mental states and the entire dynamic logic process (taking ~500 ms) are unconscious. Only the final near-logical crisp representation matching sensory percepts is available to consciousness. Most of the brain's operations (more than 99%) are inaccessible to subjective consciousness. The mind operates with “islands” of conscious-logical states in an ocean of unconsciousness; it “jumps” among conscious-logical islands over an ocean of unconscious states. And all the while we remain subjectively convinced that we are conscious. Since consciousness deals only with logical states, it is biased toward logic. For thousands of years logic has occupied a privileged position in our understanding of the mind's operations. This might explain why, after Gödel's publications received wide recognition, logic still occupies a firm place in artificial intelligence, modeling the mind, and in psychology.

## Discussion

A popular machine learning approach is statistical learning theory (SLT, Vapnik, 1999). Unlike dynamic logic, it is not related to the cognitive mechanisms of the brain-mind. Its similarity to dynamic logic is in taking on the problem of complexity. Many problems successfully solved by dynamic logic, an improvement by orders of magnitude over all other known solutions (Perlovsky et al., 2011), cannot be approached by SLT; direct comparisons for other problems have not been published. In his book Vapnik emphasized logic, relating reality and rationality. Does the SLT idea of iteratively finding support vectors overcome Gödelian limitations of logic and related complexity? This might be an interesting topic for future research. The idea of a provably self-improving algorithm has been resurrected recently by J. Schmidhuber (e.g., Steunebrink and Schmidhuber, 2012). However, other interesting ideas by this author seem to be reduced to logic and combinatorial complexity in their implementation approaches. It remains to be seen if the complexity difficulty will be overcome in future.

An exciting parallel with dynamic logic is explored in Wrede et al. (2012). These authors suggest that in the initial stage of learning, before having compositional ability, infants use a vague hierarchy with teleological representation of the final goal. Even if detailed compositional understanding is not yet available, having a vague representation of the final goal enables them to learn quickly, significantly reducing complexity of choices.

Certain principles of Gestalt psychology are confirmed in contemporary neuroscience. They are mathematically modeled by dynamic logic. For example, top-down and bottom-up signal interaction is reminiscent of a Gestalt idea (that objects in their entirety are perceived before their parts). Gestalt goals to maintain stable percepts in a noisy world are modeled via models-representations. However, these ideas are not specific to dynamic logic. Dynamic logic has modeled them mathematically, overcoming the problem of complexity.

Dynamic logic is computable. Operations used by computers implementing dynamic logic algorithms are logical. But these logical operations are at a different level than human thinking. Compare the text of this article as stored in your computer and the related computer operations to the human understanding of this article. The computer's operations are logical, but on a different level from your “logical” understanding of this article. A computer does not understand the meaning of this article the way a human reader does. The reader's logical understanding is on top of 99% of the brain's operations that are not “logical” at this level. Our logical understanding is an end state of many illogical and unconscious dynamic logic processes.

The mind's “first principles” do not include logic. Nature uses different “first principles” at its different levels of organization. Thermodynamics is not based on Newton's laws, and this was a subject of special fascination to Einstein, who emphasized that thermodynamics is a physical science with its own first principles defined at an intermediate level of organization (Einstein, 1967). It is interesting to note that Aristotle, the inventor of logic, did not use logic in his theory of the mind (forms; Aristotle, 1995). In his theory, forms are dynamic entities that evolve from vague states to crisp states in the process of “mind meeting matter” that today we call matching top-down and bottom-up signals.
